# Association of the *OLR1* rs11053646 Polymorphism with Type 2 Diabetes Risk in Saudi Adults

**DOI:** 10.2174/0118715303483523260513110232

**Published:** 2026-07-29

**Authors:** Vishal Vennu

**Affiliations:** 1 Department of Rehabilitation Sciences, College of Applied Medical Sciences, King Saud University, 10219 Riyadh, Saudi Arabia

**Keywords:** DNA isolation, fasting blood glucose, oxidized low-density lipoprotein, single nucleotide polymorphism, population, Genomic DNA, Hardy–Weinberg equilibrium (HWE)

## Abstract

**Introduction:**

Genetic polymorphisms affecting oxidative stress and lipid metabolism contribute to type 2 diabetes (T2D). The role of oxidized low-density lipoprotein receptor 1 (*OLR1*) rs11053646 single nucleotide polymorphism (SNP) in the Saudi population remains unclear. This study aimed to assess its association with T2D risk in Saudi adults.

**Methods:**

A tertiary hospital–based case–control study was conducted among 143 Saudi adults (≥18 years) in Riyadh from November 2019 to January 2020, including 79 individuals with T2D (fasting blood glucose ≥7 mmol/L) and 64 normoglycemic controls (<5.6 mmol/L). Genomic DNA was extracted from peripheral blood, and the *OLR1* rs11053646 SNP was genotyped using PCR–RFLP. Genotype distributions were tested for Hardy–Weinberg equilibrium (HWE). Logistic regression models adjusted for age, sex, and body mass index were used to estimate odds ratios (ORs) with 95% confidence intervals (CIs).

**Results:**

Genotype distribution in controls conformed to HWE (p=0.14). The GG genotype was predominant, while the CC genotype was absent. GC frequency was slightly higher in T2D cases, but not statistically significant. Allele distributions were comparable between groups. Adjusted analysis showed no significant association with T2D risk (OR=0.41, *p*=0.14).

**Discussion:**

*OLR1* rs11053646 SNP was not significantly association with T2D risk in this Saudi cohort. The absence of the CC genotype and small sample size may have limited statistical power to detect potential associations.

**Conclusion:**

*OLR1* rs11053646 SNP does not appear to be associated with T2D risk in Saudi adults. Larger studies are required to confirm these findings.

## 1 INTRODUCTION

Type 2 diabetes (T2D) is a major global health challenge characterized by chronic hyperglycemia resulting from insulin resistance and impaired insulin secretion. The prevalence of T2D has increased markedly over the past few decades due to population aging, urbanization, sedentary lifestyles, and unhealthy dietary habits [1]. The burden of T2D is particularly high in the Middle East and North Africa region. Saudi Arabia is among the countries with the highest prevalence of T2D, largely driven by increasing rates of obesity and lifestyle changes [2, 3]. In addition to environmental factors, genetic susceptibility plays an important role in determining an individual's risk of developing T2D.

Oxidative stress and lipid peroxidation are important mechanisms involved in the pathogenesis of metabolic disorders. Oxidized low-density lipoprotein (ox-LDL) contributes to endothelial dysfunction, chronic inflammation, and metabolic dysregulation [4]. The oxidized low-density lipoprotein receptor 1 (*OLR1*) gene encodes lectin-like oxidized LDL receptor-1 (LOX-1), a scavenger receptor expressed in endothelial cells, macrophages, vascular smooth muscle cells, and adipocytes [5]. LOX-1 mediates the binding, internalization, and degradation of ox-LDL and plays a key role in oxidative stress–related inflammatory signaling pathways [6].

Activation of LOX-1 has been implicated in several cardiometabolic conditions, including atherosclerosis, hypertension, and metabolic syndrome [5, 7]. Increased expression of LOX-1 has also been observed in individuals with insulin resistance and diabetes, suggesting a potential role in metabolic disease development [6].

Single-nucleotide polymorphisms (SNPs) in the *OLR1* gene have been investigated in relation to cardiometabolic traits. Previous studies have reported associations between *OLR1* variants and metabolic syndrome, cardiovascular disease, and inflammatory responses in different populations [8, 9]. Among these variants, the rs11053646 SNP has been suggested to influence metabolic and inflammatory pathways associated with cardiometabolic disorders [8]. However, the relationship between this SNP and T2D susceptibility remains insufficiently explored, particularly in Middle Eastern populations.

Saudi Arabia represents a unique population for genetic studies due to its distinct genetic background and relatively high prevalence of consanguineous marriages, which may influence the distribution of genetic variants associated with metabolic diseases [10]. Despite the high prevalence of T2D in the country, limited studies have examined the contribution of genetic polymorphisms to diabetes susceptibility among Saudi populations.

Therefore, the present study aimed to investigate the association between the rs11053646 SNP in the *OLR1* gene and T2D risk among Saudi adults living in Riyadh. Understanding the genetic determinants of T2D in this population may contribute to improved risk assessment and provide insights into the molecular mechanisms underlying metabolic disease.

## 2 MATERIALS AND METHODS

### 2.1 Study Design

This tertiary hospital-based case–control study was conducted in Riyadh, Saudi Arabia, between November 2019 and January 2020. The study protocol was approved by the Ethics Committee of the College of Applied Medical Sciences, King Saud University (Approval No. CAMS 047-37/38). All procedures adhered to the Declaration of Helsinki, and written informed consent was obtained from all participants. The study methods were reported in accordance with the STROBE guidelines [11] and the Sex and Gender Equity in Research (SAGER) guidelines to ensure appropriate consideration and reporting of sex and gender in study design, analysis, and interpretation [12].

### 2.2 Sample Size Calculation

The total sample comprised 143 participants aged ≥18 years. Statistical power was estimated at 80% for detecting an odds ratio (OR) of 2.5 for the rs11053646 SNP, with α = 0.05. The required sample size was calculated using the Farrington–Manning score test [13]:


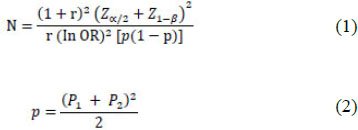


where Z_α/2_ = 1.96, α= 0.05, Z_1-β_ = 0.84, r is the sampling ratio, and p represents the event proportion in each group (p_1_ and p_2_). This calculation indicated a minimum of 31 participants per group. Our study exceeded this requirement (79 cases and 64 controls), ensuring adequate power while acknowledging that larger multicenter studies are recommended to confirm these findings.

### 2.3 Study Participants

A total of 143 Saudi adults aged ≥18 years living in Riyadh were included. Participants were divided into two groups: 79 cases diagnosed with T2D (fasting blood glucose [FBG] ≥7 mmol/L) and 64 healthy controls with normal FBG (<5.6 mmol/L) and no history of chronic disease, according to World Health Organization criteria [14]. Individuals with severe cardiac, renal, hepatic, malignant, or inflammatory diseases were excluded. Saudis living in Riyadh were selected to ensure a genetically homogenous sample, reducing type II error. Five milliliters of peripheral blood were collected in 1.5 mg/mL ethylenediamine tetraacetic acid (EDTA) tubes for biochemical and genetic analyses.

### 2.4 Biochemical Measurements

Using 3 mL of EDTA blood, FBG levels were measured with an automated clinical chemistry analyzer (COBAS system, Roche Diagnostics, Switzerland). Serum lipid profile parameters, including total cholesterol (TC), triglycerides (TG), high-density lipoprotein cholesterol (HDL-C), and low-density lipoprotein cholesterol (LDL-C), were measured using standard enzymatic methods with commercially available kits. Body mass index (BMI) was calculated as weight in kilograms divided by the square of height in meters (kg/m^2^). Height and weight were measured twice using a standardized stadiometer and a calibrated weighing scale. Participants were assessed without shoes, wallets, or heavy jewelry and wore lightweight clothing. Height was recorded to the nearest millimeter, and weight was measured to ensure accuracy and consistency.

### 2.5 DNA Extraction and Genotyping

Genomic DNA was extracted from 2 mL of EDTA-anticoagulated whole blood using a commercial genomic DNA extraction kit (Thermo Scientific, Waltham, MA, USA) following the manufacturer’s instructions. DNA purity and concentration were assessed using a NanoDrop spectrophotometer and 1% agarose gel electrophoresis. All blood samples were stored at −80 °C until analysis.

Genotyping of the *OLR1* rs11053646 polymorphism was performed using polymerase chain reaction–restriction fragment length polymorphism (PCR-RFLP) analysis. PCR amplification was carried out in a 50 µL reaction containing 60 ng of genomic DNA, 2 µL of each primer (forward: GGCTCATTTAACTGGGAAAG; reverse: CCTGTCCGTCCAAGGTCATA), 25 µL PCR master mix (Thermo Scientific), and nuclease-free water. The thermocycling protocol included initial denaturation at 95 °C for 10 min, followed by 40 cycles of denaturation at 95 °C for 30 s, annealing at 64 °C for 30 s, and extension at 72 °C for 1 min, with a final extension at 72 °C for 10 min.

The 243 bp PCR products were digested with the Fnu4HI restriction enzyme, separated on 2.5% agarose gel stained with ethidium bromide, and visualized under UV illumination. About 10% of samples underwent repeat genotyping for quality control, necessitating 100% concordance to ensure accuracy. Table **1** summarizes the SNP details.

### 2.6 Covariates

The variables included were age, sex, BMI, and lipid profile parameters such as TC, TG, HDL-C, and LDL-C. Sex was dichotomized into male and female.

### 2.7 Statistical Analysis

Continuous variables were expressed as mean ± standard deviation (SD), and categorical variables were expressed as frequencies and percentages. Normality was assessed using the Shapiro–Wilk test. Differences between cases and controls were analyzed using independent Student’s t-tests for continuous variables and chi-square tests for categorical variables.

Genotype and allele frequencies were evaluated for Hardy–Weinberg equilibrium (HWE) in the control group. Metabolic parameters (BMI, TC, TG, HDL-C, LDL-C) were compared across genotypes using an analysis of variance (ANOVA) test. Associations between the *OLR1* rs11053646 genotype (GG *vs*. GC) and T2D risk were estimated using multivariate logistic regression adjusted for age, sex, and BMI, as commonly applied in genetic association studies of cardiometabolic traits [15]. Additionally, a sensitivity subgroup analysis by sex (male and female) was performed to assess the association between the rs11053646 SNP in the *OLR1 gene* and T2D risk. OR with 95% confidence intervals (CI) were used to indicate the strength of the association. OR<1 denotes a protective or negative association, OR>1 denotes a contributing risk factor, and OR=1 denotes a null association between the disease and a certain genotype or allele variation. Due to the absence of the CC genotype, dominant and allelic models were used. Sensitivity analysis by sex was conducted to assess effect modification.

All statistical analyses were performed using SPSS version 22.0 (SPSS Inc., Chicago, IL, USA). A p-value <0.05 was considered statistically significant.

## 3 RESULTS

### 3.1 Participant Characteristics

A total of 143 participants were included in the study, comprising 79 individuals with T2D (55.2%) and 64 healthy control volunteers (44.8%). The mean age of individuals with T2D was significantly higher than that of controls (56.0 ± 11.5 *vs*. 46.2 ± 8.7 years; p < 0.001). There were no significant differences in sex distribution or BMI between the two groups. However, individuals with T2D had significantly higher levels of TC, TG, and LDL-C. HDL-C levels were significantly lower among individuals with T2D (Table **2**).

### 3.2 Association of *OLR1* rs11053646 Genotypes and Alleles with T2D

The genotype and allele distributions of the *OLR1* rs11053646 polymorphism in cases and controls are presented in Table **3**. The G allele was significantly less frequent among individuals with T2D (93%) compared with controls (96.9%). The G allele was not significantly associated with T2D risk (OR=0.47, 95% CI: 0.14–1.59; *p* = 0.22) after adjustment for age, sex, and BMI. Similarly, the GG genotype was less common in cases (86.1%) than in controls (93.8%). The GG genotype was not significantly associated with T2D risk compared with the GC genotype (OR=0.41, 95% CI: 0.12–1.37; p=0.14). HWE analysis showed that genotype distributions were consistent with equilibrium in both cases (χ^2^ = 0.44, *p* = 0.50) and controls (χ^2^ = 0.01, *p* = 0.87). The CC genotype was not observed in either group.

### 3.3 Association of rs11053646 with BMI and Lipid Profile Parameters

The relationship between the *OLR1* rs11053646 polymorphism and metabolic parameters is presented in Table **4**. No significant differences were observed in BMI or lipid profile parameters across allele groups (G *vs*. C) or genotype groups (GG *vs*. GC). Mean BMI values were similar between carriers of the G allele (25.2 ± 2.9 kg/m^2^) and C allele (24.9 ± 3.4 kg/m^2^) (*p* = 0.89), as well as between the GG (25.3 ± 2.8 kg/m^2^) and GC (24.8 ± 3.1 kg/m^2^) genotypes (*p* = 0.65). Likewise, TC, TG, HDL-C, and LDL-C levels did not differ significantly between allele or genotype groups (all *p* > 0.05).

### 3.4 Sex-stratified Analysis of rs11053646 Polymorphism

Among males, the G allele frequency was slightly lower in cases (92.1%) than in controls (92.5%); however, the association was not statistically significant (OR=0.95, 95% CI: 0.34–2.66; p=0.92). Similarly, the GG genotype was less frequent in male cases (84.2%) compared with controls (85%), but this difference was not significant (OR=0.94, 95% CI: 0.32–2.74; p=0.91). Among females, no significant association was observed for either the G allele (OR=0.93, 95% CI: 0.13–6.44; p=0.94) or the GG genotype (OR=0.90, 95% CI: 0.11–7.01; p=0.92). Sex-stratified analysis did not reveal statistically significant associations between the rs11053646 polymorphism and T2D risk in either males or females. Both genotype and allele distributions were comparable between cases and controls within each sex group (Table **5**).

## 4 DISCUSSION

This study investigated the association between the rs11053646 SNP in the *OLR1* gene and the risk of T2D among Saudi adults. The GG genotype was the most frequent, and the CC genotype was absent, while the GC genotype occurred at a lower frequency. No statistically significant association was observed between the G allele or GG genotype and T2D risk, likely due to the limited sample size and the absence of the CC genotype. These findings suggest that further studies with larger cohorts are needed to clarify the potential contribution of *OLR1* genetic variation to diabetes susceptibility in this population.

The *OLR* gene encodes LOX-1, a key receptor responsible for the binding and internalization of ox-LDL in endothelial cells, macrophages, and vascular smooth muscle cells. LOX-1 activation has been linked to oxidative stress, endothelial dysfunction, and inflammatory signaling pathways that are implicated in metabolic disorders, including insulin resistance and T2D [16, 17]. Increased circulating ox-LDL and LOX-1 expression have been reported in individuals with metabolic syndrome and diabetes, suggesting a mechanistic role for this pathway in metabolic dysregulation [18, 19].

Although the rs11053646 variant has been previously studied in the context of cardiovascular and metabolic disorders, evidence regarding its role in diabetes remains limited. A study in Chinese adults reported associations between polymorphisms in LOX-1–related pathways and diabetes risk in hypertensive patients [20]. Similarly, genetic variants in *OLR1* have been associated with cardiometabolic traits and atherosclerotic risk factors in several populations [21, 22]. Our study contributes to this body of work by reporting the genotype and allele distribution of rs11053646 in a Saudi population, providing baseline data for future investigations of its potential role in T2D susceptibility.

The biological mechanism linking *OLR1* polymorphisms to T2D may involve oxidative stress–mediated metabolic disturbances. The binding of ox-LDL to LOX-1 triggers the activation of pro-inflammatory signaling pathways and promotes the production of cytokines, matrix metalloproteinases, and adhesion molecules, which contribute to endothelial dysfunction and chronic inflammation [17]. Chronic oxidative stress and inflammatory responses are widely recognized as key contributors to insulin resistance and the pathogenesis of T2D [23]. Therefore, genetic variants affecting LOX-1 expression or activity could influence susceptibility to metabolic disease.

In the present study, no significant associations were observed between the rs11053646 genotype and metabolic parameters, such as BMI and lipid profile. Similar findings have been reported in studies examining other *OLR1* polymorphisms, which show that genetic variants influence disease susceptibility independently of conventional metabolic markers [9]. This suggests that the effect of the rs11053646 variant may be mediated through oxidative stress or inflammatory pathways rather than through direct effects on lipid metabolism. Similar findings have been reported for other genetic polymorphisms in T2D, where variants influenced disease susceptibility independently of conventional metabolic markers, possibly through oxidative stress or inflammatory pathways [24].

There is an urgent need to study gene–lifestyle interactions in Arab populations due to the high prevalence of metabolic diseases. Few studies have examined how genetic variants interact with diet or physical activity in these populations compared to other ethnic groups [25]. Additionally, in the Saudi population, metabolic parameters (BMI, TC, TG, HDL-C, LDL-C) show little association with genotype. Investigating these interactions can provide important insights into the impact of T2D on metabolic health in Saudi Arabia.

Sex-stratified analyses did not reveal statistically significant associations between genotype and T2D risk in either males or females, although the frequency of the G allele was higher in cases in both groups. Previous studies have reported sex-specific associations between *OLR1* polymorphisms and cardiovascular traits [26]. The lack of significant sex-specific effects in our study may be due to the limited sample size.

To our knowledge, this study is the first to examine the *OLR1* rs11053646 polymorphism in relation to T2D risk in Saudi adults. While no statistically significant association was observed, these findings provide preliminary data that complement prior genetic studies in this population, which identified links between other polymorphisms and T2D susceptibility [27, 28]. The Saudi population is particularly important for genetic research due to its unique population structure and high prevalence of consanguineous marriages, which may affect the distribution of metabolic disease–related genetic variants [29].

Nevertheless, understanding genetic contributions to T2D remains important given the high prevalence of T2D and obesity in Saudi Arabia. Although no significant association was observed for the *OLR1* rs11053646 polymorphism in this study, identifying genetic variants linked to metabolic disease may ultimately improve risk assessment and guide personalized prevention strategies. The Saudi population has a relatively limited genetic structure [30] and remains genetically distinct from other Arab regions [29] due to high rates of consanguineous marriages and family history of diabetes, which can increase T2D risk in offspring [31]. Saudi Arabia ranks second in the Middle East for obesity and third for T2D prevalence [32]. Consanguineous marriages are relatively common in Saudi Arabia, with prevalence rates exceeding 50% in some regions, and are generally higher than or comparable to those reported in neighboring Middle Eastern and other global populations [33-35]. These factors contribute to a high incidence of T2D, particularly among individuals from consanguineous unions, which may increase genetic susceptibility to metabolic disorders [31]. This trend places a substantial burden on public health and healthcare systems in Saudi Arabia [1, 2]. This situation underscores the urgent need for effective and preventive strategies to reduce the burden of T2D, in line with the national public health priorities outlined in Saudi Vision 2030 [2, 36].

### 4.1 Study Limitations

Several limitations should be considered when interpreting these findings. First, the study included a relatively small sample size, which may reduce statistical power and lead to wide confidence intervals. This limitation may affect both the internal and external validity of the results and, therefore, warrants cautious interpretation. Second, only a single SNP (rs11053646) in the *OLR1* gene was examined, which may not fully capture the genetic variability at this locus associated with T2D susceptibility. Third, the absence of individuals carrying the CC genotype limited genotype comparisons and prevented evaluation of recessive genetic models.

Moreover, all participants were recruited from a single tertiary hospital in Riyadh, which may introduce selection bias and limit the generalizability of the findings to the broader Saudi Arabian population. In addition, the study included only individuals of Saudi ethnicity, which further restricts extrapolation to other ethnic groups within the region. Furthermore, some clinical variables exhibited relatively large standard deviations, reflecting variability within the sample.

Moreover, although analyses were adjusted for age, sex, and BMI, other potentially important confounding factors, such as lifestyle behaviors, dietary patterns, and family history of diabetes, were not available. In addition, subgroup analyses based on age categories, BMI groups, diabetes duration, and the presence of diabetic complications could not be conducted because of the limited sample size and incomplete availability of certain clinical variables. Only sex-based subgroup analysis was feasible. Finally, recruitment barriers in case–control studies may vary across demographic groups, including age and socioeconomic factors, highlighting the need for tailored recruitment strategies to improve representativeness and participation rates [37]. In addition, the case–control design does not allow causal inference. Future large-scale, multi-center studies are needed to validate these findings and to explore gene–phenotype interactions across diverse Saudi populations.

## CONCLUSION

This study examined the association between the *OLR1* rs11053646 SNP and T2D risk among Saudi adults. No statistically significant association was observed between rs11053646 genotypes and T2D susceptibility in the present sample. However, the absence of the CC genotype and the relatively small sample size may have limited the statistical power to detect modest genetic effects. Larger multicenter studies are required to confirm these findings and to further explore the potential role of *OLR1* genetic variation in the pathogenesis of T2D.

## AUTHORS’ CONTRIBUTIONS

The author confirms sole responsibility for the following: study conception and design, data collection, analysis and interpretation of results, and manuscript preparation.

## Figures and Tables

**Table 1:** Details of the SNP involved in this study.

**Gene**	**SNP ID**	**Variant**	**Chr.**	**PC**	**PCR**	**Enz.**	**RFLP**
*OLR1*	rs11053646	C/G	10160849	Lys-Asn	243bp	FNU4HI	G-243bp; C-222/21bp

**Abbreviations:** SNP, single nucleotide polymorphism; Chr., chromosome; PC, protein change; PCR, polymerase chain reaction; Enz. Enzyme. RFLP, restriction fragment length polymorphism.

**Table 2:** Basic characteristics of study participants.

**Characteristics**	**Cases n=79 (55.2%)**	**Controls n=64 (44.8%)**	***p-*value**
Age in years	56.03 ± 11.5	46.18 ± 8.7	<0.001^a^
**Sex, n (%)**	-	-	0.79^b^
Male	57 (72.2)	40 (62.5)	-
Female	22 (27.8)	24 (37.5)	-
BMI, kg/m^2^	25.3 ± 2.4	25.1 ± 2.9	0.61^a^
TC, mmol/L	4.9 ± 0.9	4.3 ± 1.3	0.01^a^
TG, mmol/L	1.9 ± 0.9	1.2 ± 0.5	0.01^a^
HDL-C, mmol/L	0.6 ± 0.1	0.8 ± 0.5	0.001^a^
LDL-C, mmol/L	3.4 ± 0.9	2.1 ± 0.7	0.002^a^

**Abbreviations:** BMI, body mass index; TC, total cholesterol; TG, triglycerides; HDL-C, high-density lipoprotein-cholesterol; LDL-C, Low-density lipoprotein-cholesterol. **Note:** Mean ± SD was used to provide continuous data, while frequencies and percentages were used to present categorical data. For categorical variables, a chi- squared test **^b^** was used to determine the *p-*value, and for continuous variables, an independent Student’s *t*-test **^a^** was used.

**Table 3:** Allelic and genotypic distribution and association of the rs11053646 (*OLR1*) polymorphism between cases and controls.

**Variations**	**Cases n (%)**	**Controls n (%)**	OR (95% CI)*	***p*-value**
Alleles	-	-	-	-
G allele	147 (93.0)	124 (96.9)	0.47 (0.14–1.59)	0.22
C allele	11 (7.0)	4 (3.1)	Reference	-
Genotypes	-	-	-	-
GG	68 (86.1)	60 (93.8)	0.41 (0.12–1.37)	0.14
GC	11 (13.9)	4 (6.2)	Reference	-
CC	0	0	-	-
HWE analysis	χ^2^ = 0.44, *p* = 0.50	χ^2^ = 0.01, *p* = 0.87	-	-

**Abbreviations:** OR, odds ratios; CI, confidence interval; HWE, Hardy-Weinberg equilibrium. **Note:** Data are presented as frequencies and percentages. Genotype and allele frequencies were determined using the HWE analysis. Logistic regression was used to present the OR and 95% CI. *Adjusted for age, sex, and BMI.

**Table 4:** Association of the rs11053646 (*OLR1*) polymorphism with BMI and lipid profile parameters.

**Covariates**	**G**	**C**	***p*-value**	**GG**	**GC**	***p*-value**
BMI, kg/m^2^	25.2 ± 2.9	24.9 ± 3.4	0.89	25.3 ± 2.8	24.8 ± 3.1	0.65
TC, mmol/L	5.9 ± 0.7	5.2 ± 1.2	0.23	5.1 ± 0.9	5.0 ± 1.1	0.22
TG, mmol/L	1.8 ± 1.0	1.5 ± 1.0	0.42	1.7 ± 1.0	1.6 ± 0.6	0.31
HDL-C, mmol/L	0.6 ± 0.2	0.5 ± 0.2	0.45	0.7 ± 0.2	0.6 ± 0.2	0.42
LDL-C, mmol/L	3.6 ± 0.9	3.5 ± 0.8	0.54	3.6 ± 0.8	3.7 ± 0.9	0.73

**Abbreviations:** BMI, body mass index; TC, total cholesterol; TG, triglycerides; HDL-C, high-density lipoprotein-cholesterol; LDL-C, Low-density lipoprotein-cholesterol. **Note:** Data presented in Mean ± SD.

**Table 5:** Sex-specific association of the rs11053646 (*OLR1*) alleles and genotypes between cases and controls.

**Variations**	**Male Cases n (%)**	**Male Controls n (%)**	OR (95% CI)*	**p-value**	**Female Cases n (%)**	**Female Controls n (%)**	OR (95% CI)*	***p*-value**
**Alleles**	-	-	-	-	-	-	-	-
**G allele**	105 (92.1)	74 (92.5)	0.95 (0.34–2.66)	0.92	42 (95.5)	46 (95.8)	0.93 (0.13–6.44)	0.94
**C allele**	9 (7.9)	6 (7.5)	Reference	-	2 (4.5)	2 (4.2)	Reference	-
**Genotypes**	-	-	-	-	-	-	-	-
**GG**	48 (84.2)	34 (85.0)	0.94 (0.32–2.74)	0.91	20 (90.9)	22 (91.7)	0.90 (0.11–7.01)	0.92
**GC**	9 (15.8)	6 (15.0)	Reference	-	2 (9.1)	2 (8.3)	Reference	-
**CC**	0	0	-	-	0	0	-	-

**Abbreviations:** OR, odds ratios; CI, confidence interval; HWE, Hardy-Weinberg equilibrium. **Note:** Data are presented as frequencies and percentages. Genotype and allele frequencies were determined using the HWE analysis. Logistic regression was used to present the OR and 95% CI. *Adjusted for age, sex, and BMI.

## Data Availability

The data used and/or analyzed during this study are available from the corresponding author upon reasonable request.

## LIST OF ABBREVIATIONS

T2D: Type 2 Diabetes
BMI: Body Mass Index
FBG: Fasting Blood Glucose
WC: Waist Circumference
HC: Hip Circumference
TC: Total Cholesterol
TG: Triglycerides
HDL-C: High-Density Lipoprotein Cholesterol
LDL-C: Low-Density Lipoprotein Cholesterol
SNP: Single Nucleotide Polymorphism
OLR1: Oxidized Low-Density Lipoprotein Receptor 1
LOX-1: Lectin-Type Oxidized LDL Receptor 1
sLOX1: Soluble Lectin-Like Oxidized LDL Receptor-1
PCR: Polymerase Chain Reaction
PCR-RFLP: Polymerase Chain Reaction–Restriction Fragment Length Polymorphism
EDTA: Ethylenediaminetetraacetic Acid
DNA: Deoxyribonucleic Acid
HWE: Hardy–Weinberg Equilibrium
OR: Odds Ratio
CI: Confidence Interval
ox-LDL: Oxidized Low-Density Lipoprotein
MetS: Metabolic Syndrome
MMPs: Matrix Metalloproteinases

## STANDARDS OF REPORTING

STROBE and SAGER guidelines were followed.
